# Oxidation of sulfur, hydrogen, and iron by metabolically versatile *Hydrogenovibrio* from deep sea hydrothermal vents

**DOI:** 10.1093/ismejo/wrae173

**Published:** 2024-09-14

**Authors:** Katja Laufer-Meiser, Malik Alawi, Stefanie Böhnke, Claus-Henning Solterbeck, Jana Schloesser, Axel Schippers, Philipp Dirksen, Thomas Brüser, Susann Henkel, Janina Fuss, Mirjam Perner

**Affiliations:** Marine Geosystems, GEOMAR Helmholtz Centre for Ocean Research Kiel, Wischhofstraße 1-3, 24148 Kiel, Germany; Bioinformatics Core, University Medical Center Hamburg-Eppendorf, Martinistrasse 51, 20246 Hamburg, Germany; Marine Geosystems, GEOMAR Helmholtz Centre for Ocean Research Kiel, Wischhofstraße 1-3, 24148 Kiel, Germany; Institute for Materials and Surfaces, Kiel University of Applied Sciences, Grenzstrasse 3, 24149 Kiel, Germany; Institute for Materials and Surfaces, Kiel University of Applied Sciences, Grenzstrasse 3, 24149 Kiel, Germany; Federal Institute for Geosciences and Natural Resources (BGR), Stilleweg 2, 30655 Hannover, Germany; Bioinformatics Core, University Medical Center Hamburg-Eppendorf, Martinistrasse 51, 20246 Hamburg, Germany; Institute of Microbiology, Leibniz Universität Hannover, Herrenhäuser Straße 2, 30419 Hannover, Germany; Alfred Wegener Institute Helmholtz Centre for Polar and Marine Research, Am Handelshafen 12, 27570 Bremerhaven, Germany; Institute of Clinical Molecular Biology ,Kiel University, Rosalind-Franklin-Straße 12, 24105 Kiel, Germany; Marine Geosystems, GEOMAR Helmholtz Centre for Ocean Research Kiel, Wischhofstraße 1-3, 24148 Kiel, Germany

**Keywords:** *Hydrogenovibrio*, iron oxidizer, hydrogen oxidizer, sulfur oxidizer, chemolithoautotrophy, autotrophic CO_2_ fixation: Indian ridge, hydrothermal vent environment bacteria

## Abstract

Chemolithoautotrophic *Hydrogenovibrio* are ubiquitous and abundant at hydrothermal vents. They can oxidize sulfur, hydrogen, or iron, but none are known to use all three energy sources. This ability though would be advantageous in vents hallmarked by highly dynamic environmental conditions. We isolated three *Hydrogenovibrio* strains from vents along the Indian Ridge, which grow on all three electron donors. We present transcriptomic data from strains grown on iron, hydrogen, or thiosulfate with respective oxidation and autotrophic carbon dioxide (CO_2_) fixation rates, RubisCO activity, SEM, and EDX. Maximum estimates of one strain’s oxidation potential were 10, 24, and 952 mmol for iron, hydrogen, and thiosulfate oxidation and 0.3, 1, and 84 mmol CO_2_ fixation, respectively, per vent per hour indicating their relevance for element cycling *in-situ*. Several genes were up- or downregulated depending on the inorganic electron donor provided. Although no known genes of iron-oxidation were detected, upregulated transcripts suggested iron-acquisition and so far unknown iron-oxidation-pathways.

## Introduction

In deep-sea hydrothermal vent systems, hot and reduced hydrothermal fluids enriched in carbon dioxide (CO_2_), hydrogen (H_2_), methane, reduced sulfur (S) species, and metals (e.g., iron, Fe), rise from cracks in the basaltic ocean crust where seawater is circulating and transformed into hydrothermal fluids. They mix with entrained ambient, oxic, cold waters, creating thermal and chemical gradients along the fluid pathway [1]. In the mixing zone, intermediate inorganic S species like S^0^, thiosulfate (S_2_O_3_^2−^), and polysulfide can be found [2, 3]. This chemical disequilibrium can be exploited by chemolithotrophic microorganisms catalyzing the oxidation of these reduced substances to conserve energy that can be utilized for autotrophic CO_2_ fixation [1, 4]. One group of chemolithoautotrophs that is often abundant in well mixed hydrothermal environments are *Hydrogenovibrio* spp. [5–9]. Members of this group were originally placed into the genus *Thiomicrospira* [10] and described as typical S-oxidizers capable of using H_2_, hydrogen sulfide (H_2_S), S_2_O_3_^2−^, [S_n_(SO_3_)_2_]^2−^, and S^0^ under aerobic or microaerobic conditions [7, 8, 11, 12]. Based on physiology, morphology, and phylogeny they were reclassified as *Hydrogenovibrio* [13].

Recent work showed that several *Hydrogenovibrio* spp. can harness energy from H_2_ oxidation [5, 14]. The so far only strain capable of Fe(II) and S_2_O_3_^2−^ oxidation, *Hydrogenovibrio* sp. SC-1, was isolated from a non-hydrothermal marine habitat by microbial traps positioned in Catalina Island off California, USA [15]. To our current knowledge, Fe(II) oxidation has not been tested for other *Hydrogenovibrio* spp. However, in incubation experiments with hydrothermal fluids to which Fe(II) was added, CO_2_ fixation rates were highly stimulated, while the relative abundance of *Hydrogenovibrio/Thiomicrospira* increased from 15% in the environment to >50% in the incubations [6], highlighting that this metabolic potential is likely more common in this group than thought. Until today it has not been demonstrated that a member of this group can grow with all three electron donors (Fe(II), H_2_, S). Such metabolic versatility would be highly beneficial, given the temporal and spatial dynamics existing in hydrothermal vent environments and may explain why *Hydrogenovibrio* species are present at geographically distinct places with distinct vent chemistry [14, 16].

Here we report the isolation of three *Hydrogenovibrio* strains from three different vents along the Indian Ridge (IR). Members of the genus *Hydrogenovibrio* have previously been shown to dominate in hydrothermal vent communities of the South-West Indian Ridge (SWIR) [9, 17], and more recently a strain has been isolated from an active hydrothermal vent chimney at the SWIR that is capable of S and H_2_ oxidation (*Hydrogenovibrio thermophilus* strain S5) [14]. We demonstrate that our isolates can oxidize Fe(II), H_2_, and S_2_O_3_^2−^, thereby gaining sufficient energy to synthesize biomass. We calculate the potential these strains have for element cycling in IR vents. Further, we link this physiological work with shifts in the transcriptomic data under the three incubation conditions and identify genes related to the different metabolisms.

## Material and methods

### Field sites and sampling

Hydrothermal vent fluid samples were collected during the BGR led INDEX 2019 cruise on RV Sonne SO-271 along the Central Indian Ridge (CIR) and South-East Indian Ridge (SEIR). Field sites, sampling, and chemical parameters of the hydrothermal fluids are described in detail by Adam et al. [17]. Fluid samples used for the isolation of the three strains were 040 KIPS C/D (F1, VF4, CIR), 083 KIPS A/B (F2, VF1, SEIR), 104 KIPS C/D (F3, VF2, SEIR) (for more details see Table S4).

### Quantification of cell numbers

For cell counts, a subsample of the hydrothermal fluids was fixed with 4% formaldehyde) for 24 h at 4°C. The fixed cells were then concentrated on polycarbonate filters (type: Nucleopore, 0.2 μm pore size, Whatman, Buckinghamshire, United Kingdom), washed with sterile PBS and stored at −20°C. Filter sections were stained with DAPI (4′,6-Diamidin-2-phenylindol) and cells counted under an epifluorescence microscope.

### Enrichment and isolation

Alongside the enrichment cultures that were described by Adam et al. [17], enrichments from the fluids 040 KIPS C/D, ROPOS 083 KIPS A/B, and 104 KIPS C/D were set up on ZVI (zero valent iron) plates under microoxic conditions to enrich for microaerophilic Fe(II)-oxidizers. The ZVI plates were prepared as previously described [18] with artificial seawater (ASW), N_2_/CO_2_ (80:20) as headspace and pH 6.8. Detailed information on the ZVI plates and medium composition can be found in the SI (Table S3). After the initial enrichment, cultures were subjected to dilution to extinction series for isolation. Growth was regularly checked by brightfield and fluorescence microscopy, where the cells were stained with LIVE/DEAD stain (BacLight, Invitrogen, Waltham, MS, USA). The purity of the culture was confirmed by microscopic examination and sequencing of the 16S rRNA gene. Strains 083 and 104 were deposited at the DSMZ under accession numbers DSM 117350 and DSM 117349. Strain 040 was physiologically very similar to strain 104 and therefore not deposited at the DSMZ.

### DNA isolation and sequencing of the 16S rRNA gene

DNA was extracted with the NucleoSpin Soil kit (Macherey-Nagel, Düren, Germany) according to the manufacturer’s instructions. The bacterial 16S rRNA gene was PCR amplified using the primers 27F/1492R [19] and sequenced by Sanger sequencing (Eurofins Genomics, Ebersberg, Germany). The phylogenetic tree of the strains and their closest relatives was constructed with MEGA X [20] based on the Maximum-likelihood method and Tamura-Nei model with 1000 bootstrap replications after multiple alignments using ClustalW [21].

### Growth on various electron acceptors and donors

Growth was tested on FeS (in gradient tubes) and FeCl_2_ under microoxic conditions, FeCl_2_ and NO_3_^−^, H_2_S and NO_3_^−^, H_2_S, and O_2_ (in gradient tubes), S_2_O_3_^2−^ and NO_3_^−^, and S_2_O_3_^2−^ and O_2_.

#### Growth on Fe(II) and quantification of Fe(II) oxidation rates

To confirm growth on Fe(II) cultures were also grown in gradient tubes with an FeS plug in the bottom and in liquid ASW medium containing FeCl_2_. Gradient tubes contained gel-stabilized ASW with a bottom layer containing 1% (wt/vol) agarose with a 1:1 mixture of FeS and ASW medium overlain by ASW medium with 0.15% (wt/vol) agarose and air in the headspace [22, 23]. In gradient tubes, cultures were inoculated vertically over the whole length of the tube. Cultures in ASW with FeCl_2_ were grown in 100 ml serum vials containing 50 ml ASW, 500 μM FeCl_2_ with a headspace of N_2_/CO_2_ (80:20) to which sterile air was added to reach an oxygen concentration of ~1%. The headspace was flushed daily with N_2_/CO_2_, and afterwards 500 μM FeCl_2_ and fresh sterile air (1% final O_2_ concentration) was added.

Fe(II) oxidation rates were determined in cultures grown in ASW with FeCl_2_. Each day samples from the culture were taken and fixed with 1 M HCl (final concentration). At the same time, a sample for cell counting was taken and fixed with 4% formaldehyde. The concentration of Fe(II) and total Fe was measured photometrically with the Ferrozine Assay [24], and for total Fe measurements all Fe(III) was reduced to Fe(II) with hydroxylamine hydrochloride. Fe(III) concentrations were calculated by subtracting Fe(II) from total Fe. Uninoculated replicates were taken as abiotic controls. Fe(II) oxidation rates were calculated from the difference between the biotic and abiotic incubations. Total cell counts were performed as described above.

#### Growth on H_2_ and quantification of H_2_ oxidation rates

Cultures growing on H_2_ were cultivated in 16 ml Hungate tubes, containing MJ medium (detailed composition Table S3) with a H_2_/CO_2_/O_2_ (79:20:1) headspace. Resazurin was added as redox indicator and the cultures were flushed with H_2_/CO_2_/O_2_ (79:20:1) regularly once the medium became clear.

For quantification of H_2_-consumption rates, cultures were grown in 100 ml serum vials with 50 ml MJ medium and a H_2_/N_2_/CO_2_/O_2_ (2:77:20:1) headspace. H_2_ concentrations in the headspace were measured with a Trace GC Ultra gas chromatograph (ThermoFisher Scientific, Waltham, MA, USA), using a ShinCarbon ST 100/120 column (Restek Corporation, Bellefonte, PA, USA) and a Pulsed Discharge Detector (Vici Valco Instruments, Houston, TX, USA) as previously described [5]. Uninoculated replicates were used as controls. Cell counts were performed as described above.

#### Growth on S_2_O_3_^2−^ and quantification of S_2_O_3_^2−^ oxidation rates

Cultures growing on S_2_O_3_^2−^ were cultivated in 100 ml serum flasks containing 50 ml of T-ASW medium with 40 mM S_2_O_3_^2−^ (detailed composition Table S3) and sterile air in the headspace. Cultures were also grown on T-ASW plates, which were prepared with T-ASW medium and 1.5% agar. For quantification of S_2_O_3_^2−^oxidation rates, samples were taken over time and the S_2_O_3_^2−^ concentration was measured via high-performance liquid chromatography (HPLC) as previously described [25, 26]. Uninoculated replicates were used as controls. Cell numbers were quantified as described above.

### Quantification of ^14^C-HCO_3_^−^ incorporation rates during Fe(II), H_2_, and S_2_O_3_^2−^ oxidation

For quantification of ^14^C-HCO_3_^−^ incorporation rates for strain 104 with the three different substrates, cultures were set up as described above for the rate measurements. The following treatments were prepared in triplicates for each of the electron donors in a ^14^C version and a no radioactivity version: (i) control without cells with electron donor, (ii) control with cells without electron donor, and (iii) a treatment with cells with electron donor. The ^14^C treatments were initially incubated and sampled in parallel with the other treatments and after 6 h for FeCl_2_, 8 h for H_2_ and 24 h for S_2_O_3_^2−^ 1 μCi of ^14^C-NaHCO_3−_ (specific activity of 50–60 mCi mmol^−1^) was injected to the vials and incubated for 18, 17, and 24 h for FeCl_2_, H_2_, and S_2_O_3_^2−^, respectively. The ^14^C incubation was stopped by adding formaldehyde (final concentration 4% (v/v)). At the same time, the non-^14^C parallel treatments were continuously sampled to determine the consumption of FeCl_2_, H_2_ and S_2_O_3_^2−^, and cell numbers during the ^14^C incubation.

For quantification of ^14^C incorporation, samples for DIC concentration measurements were taken before tracer addition, and fixed with HgCl_3_. For cultures grown on H_2_ and FeCl_2_ DIC was quantified with a QuAAtro four-channel flow injection Analyzer (Seal Analytical) and the respective standard QuAAtro method (Q-067-05 Rev.1). For cultures grown on S_2_O_3_^2−^ DIC was quantified by flow-injection using a conductivity method with 30 mM HCl as carrier and 5 mM NaOH as receiver.

Just before the ^14^C incubation was stopped, 100 μl of supernatant from each vial was added to a scintillation vial containing scintillation cocktail (Ultima Gold XR, PerkinElmer) to determine the total radioactivity in the culture by liquid scintillation counting (TriCarb 291 001, PerkinElmer). The remaining culture was concentrated on polycarbonate filters (pore size 0.22 μm), washed with sterile PBS solution, and leftover bicarbonate was removed by acid fuming for 24 h with 2 M HCl in a desiccator. The FeCl_2_ filters were additionally treated with oxalic acid/ammonium oxalate solution (100 mM/80 mM) and Fe(II)-EDAS (100 mM) while the filter was still mounted on the filtration tower to dissolve Fe-minerals, including siderite, and washed with sterile PBS again before acid fuming. The filters were added to scintillation vials, scintillation cocktail was added and the radioactivity quantified by liquid scintillation counting. The rate of C-fixation per ml culture per hour was calculated as described previously [27].

### Quantification of RubisCO enzyme activity

Specific RubisCO activity was measured with an HPLC based enzyme assay as previously described [28, 29]. A 1 l batch of *Hydrogenovibrio* strain 104 was cultured under the same conditions as outlined above for the rate measurements with H_2_. Crude extracts were prepared using a French pressure cell press (Thermo Spectronic) (for detailed information see SI). RubisCO activity was measured at 25°C in RubisCO assay buffer [100 mM Tris–HCl (pH 7.8), 10 mM MgCl_2_, 1 mM EDTA, 25 mM NaHCO_3_, and 1 mM DTT] with 0.2 mg μl^−1^ protein crude extract and 10 mM ribulose-1,5-bisphosphate (RuBP). The consumption of RuBP was quantified using the LaChrom Elite HPLC system (Hitachi, Tokio, Japan) with a Lichrospher 100 RP 18e column (VWR International GmbH, Darmstadt, Germany) (for further information see SI).

### Transcriptomic analyses under different growth conditions

For transcriptomic analyses, strain 104 was grown with H_2_, S_2_O_3_^2−^, and FeCl_2_ as described above (45 × 50 ml). After harvesting the cultures in the late exponential phase by centrifugation, RNA was extracted using the Marchery and Nagel NucleoBond RNA soil mini kit (Düren, Germany) according to manufacturer’s protocol but with Chloroform:Isoamylalcohol (24:1) instead of Phenol:Chloroform:Isoamylalcohol (25:24:1). Residual genomic DNA was removed using the RapidOut DNA Removal Kit as specified by the manufacturer (ThermoFisher Scientific) and the absence of genomic DNA was verified via PCR. Library preparation for transcriptome sequencing was done with the Stranded Total RNA Prep kit with the Ribo-Zero plus rRNA depletion kit (Illumina, San Diego, USA) according to manufacturer’s protocol. Sequencing was performed on a NovaSeq 6000 System (2 × 150 bp; Illumina) with the NovaSeq 6000 SP Reagent Kit v1.5 and NovaSeq XP 2-Lane Kit v1.5.

Sequence reads were processed with FASTP (v0.23.2) [X] to remove artificial sequences originating from adapters, low quality sequences (more than 40% of bases below a phread quality score below 15), and to correct bases in the overlap regions of the forward and reverse reads of each read pair. Reads were than aligned to the NCBI RefSeq assembly GCF_003991075.1 (strain S5, which had based on 16S rRNA gene 100% sequence similarity to our strain) with BWA MEM (v0.7.17-r1188). Samtools (v1.16) was used for sorting the alignments and featureCounts from the Subread package (v.2.0.6) was employed to obtain counts per gene based on the genomic coordinates provided with the aforementioned RefSeq assembly. Normalization and differential expression analysis were carried out with DESeq2 (v.1.40.1). Although the selection of the reference strain can have a significant impact on the results, we also carried out a complementary analysis without using any reference strain. For this, we performed a transcriptome assembly with Trinity (v2.15.1) and then annotated the assembled transcripts with Prokka (v1.14.6). By this approach we could also not detect any of the known genes involved in Fe oxidation (e.g., cyc2) as summarized elsewhere [30].

## Results

### Isolation of three *Hydrogenovibrio* strains from IR vent systems

**Fig. 1:**
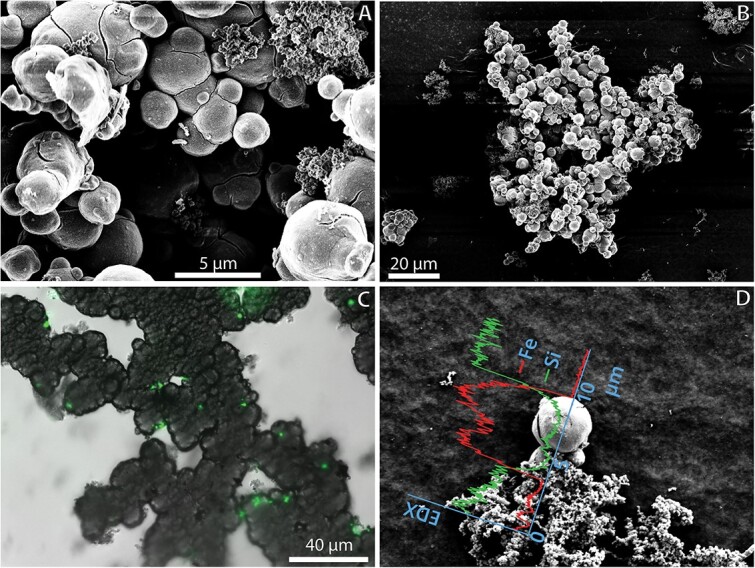
Microscopy, and EDX of culture 104 grown on ZVI. (A and B) SEM images. In a cells attached to the bulbous mineral structures can be seen. (C) Overlay of brightfield and fluorescence microscopy. The Fe-minerals appear dark in brightfield microscopy, the cells are stained with LIVE/DEAD stain and visible in fluorescence microscopy. D: SEM image with overlain EDX spectrum measured along the x-axis of the EDX graph.

**Fig. 2:**
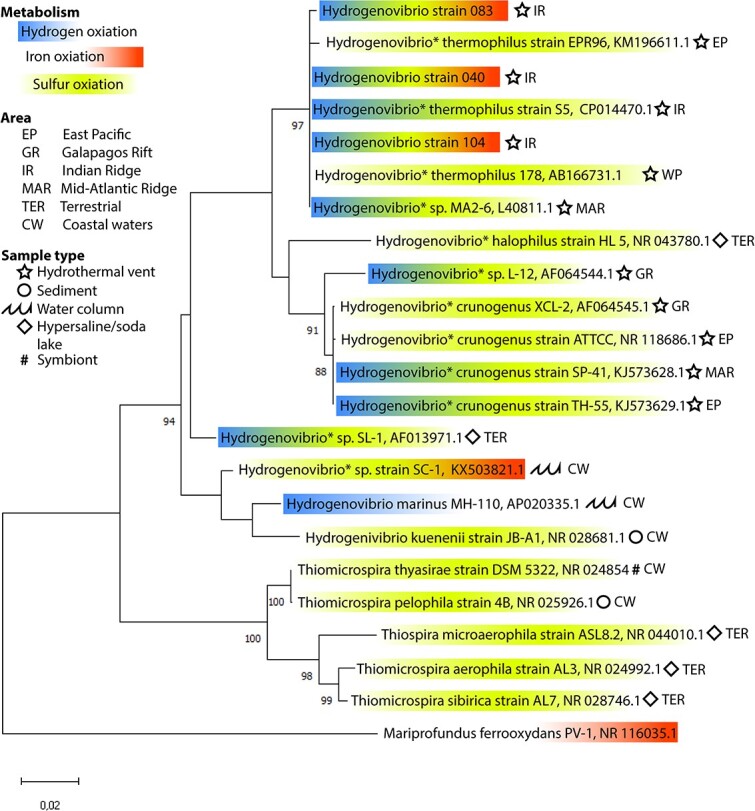
Phylogenetic tree. Phylogenetic tree based on 16S rRNA gene sequences, showing the phylogenetic relationship of the three isolates and closely related strains. ^*^ indicate which strains have been reclassified from *Thiomicrospira* to *Hydrogenovibrio* [13]. Colors indicate experimentally verified metabolic capabilities of the strains as indicated in the legend. Bootstrap values are only displayed if above 80%. The scale bar represents the expected number of changes per nucleotide position. *M. ferrooxydans* is added as an outgroup.

Hydrothermal fluids from VF4 (CIR), VF1 (SEIR), and VF2 (SEIR) [17] were used for enrichment on ZVI-plates. Cells were uniform and were mostly attached to Fe-minerals, which appeared as bulbous structures. EDS analysis confirmed that the bulbous structures consist of elemental Fe (Fig. 1) and XRD analysis of the products confirmed existence of crystalline α-Fe. 16S rRNA gene sequence comparison showed that the strains were most closely related to *Hydrogenovibrio thermophilus* strains JR-2 and S5 (Fig. 2) with 99.9, 100, and 100% sequence similarity for strain 040, 083, and 104, respectively. More information can be found in SI.

### Growth of strains and quantification of electron donor oxidation and C-fixation rates

Growth was observed on FeS gradient tubes, FeCl_2_ and O_2_ (microoxic, 1% O_2_), H_2_ and O_2_ (microoxic, 1% O_2_), and S_2_O_3_ [2] and O_2_ (fully oxic) (Table 1). On H_2_ (MJ medium) and S_2_O_3_^2−^ (T-ASW medium), growth was confirmed by color change of the medium (blue to pink to colorless for MJ and red to yellow for T-ASW) as well as by microscopy. On T-ASW, growth was also visible from the formation of a white precipitate, likely ZVS (zero valent S).

**Table 1:** Effect of redox substrates on growth of strains 040, 083, and 104.

	e^−^ acceptor	
e^−^ donor	O_2_	NO_3_^−^
ZVI	+	n.d.
FeCl_2_	+	−
FeS gradient	+	n.d.
H_2_	+	n.d.
H_2_S	−	−
S_2_O_3_^2−^	+	n.d.

n.d. = not determined

#### Microbial Fe(II), H_2_, and S_2_O_3_^2−^ oxidation rates

**Fig. 3:**
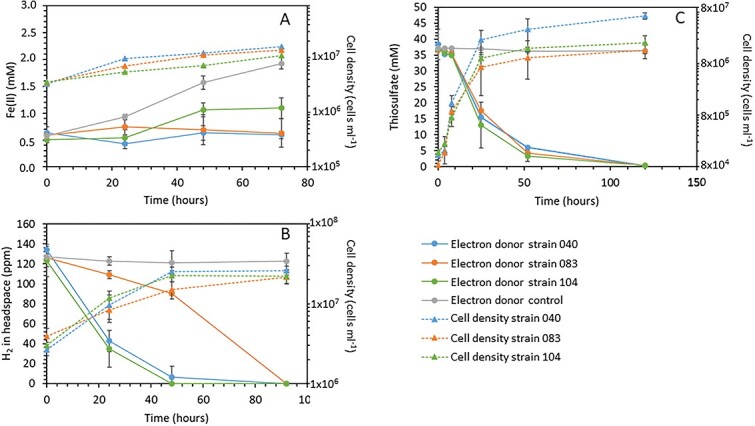
Rates of electron donor oxidation and cell numbers over time. Concentrations of electron donors and numbers of cells over time for all three cultures grown on (A) Fe(II), (B) H_2_^−^, and (C) S_2_O_3_^2−^. Symbols are the mean (n = 3), error bars show standard deviation.

Cell numbers in the Fe(II)-amended cultures increased on average 4.8-, 3.9-, and 3.0-fold within 3 days for culture 040, 083, and 104. This is lower than what was reported for SC-1, for which cell numbers increased by two orders of magnitude within a week [15]. Maximum Fe(II) oxidation rates were 0.016 (± 0.002), 0.008 (± 0.004), and 0.010 (± 0.002) μmol Fe(II) ml ^−1^ h^−1^ for culture 040, 083, and 104, respectively (± are SD). Cell-specific rates were 1.6, 0.9, and 1.7 fmol Fe(II) cell^−1^ h^−1^ for culture 040, 083, and 104, respectively. Cultures 040 and 104 completely oxidized all H_2_ in the headspace within 48 h; culture 083 was slower but also completely oxidized all H_2_ available within 96 h (Fig. 3). Maximum H_2_ oxidation rates were 145.2 (± 18.2), 75.4 (±3.0), and 140.3 (±6.2) nmol ml^−1^ h^−1^ for culture 040, 083, and 104, respectively. Per cell, the rates were 1.8, 9.2, and 1.7 fmol H_2_ cell^−1^ h^−1^. This is very similar to what is reported for strain SP-41 (1.47–6.10 fmol H_2_ cell^−1^ h^−1^) [5]. In all cultures S_2_O_3_^2−^ was completely oxidized within 120 h (Fig. 3). Maximum S_2_O_3_^2−^ oxidation rates were 1.16 (± 0.026), 1.05 (± 0.05), and 2.06 (± 0.014) μmol S_2_O_3_^2−^ ml^−1^ h^−1^ for culture 040, 083, and 104, respectively. Cell specific rates were 53.6, 190.8, and 262.4 fmol S_2_O_3_^2−^ cell^−1^ h^−1^ for culture 040, 083, and 104, respectively. The oxidation rates are similar to those reported for *Hydrogenovibrio thermophilus* S5, which oxidized maximal 1.04 μmol S_2_O_3_^2−^ ml^−1^ h^−1^ [14].

#### Autotrophic CO_2_ fixation rates

Autotrophic CO_2_ fixation rates were determined for strain 104 by measuring ^14^C-HCO_3_^−^ incorporation during growth on FeCl_2_, H_2_, and S_2_O_3_^2−^. The highest rates of ^14^C-HCO_3_^−^ fixation (per ml culture and per cell) were found when the culture was grown with S_2_O_3_^2−^, whereas the lowest C-fixation rates were found when the culture was grown with Fe(II) as electron donor (Table 1). ^14^C incorporation measurements with hydrothermal fluids conducted in other studies reported significantly lower cell-specific rates in the range of 0.0001–0.1 fmol cell^−1^ h^−1^ [31, 32]. In a previous study [6] rates quantified in incubation experiments with hydrothermal fluids to which Fe(II) was added were in the range of 4 × 10^−7^ to 20 × 10^−7^ mmol C ml^−1^ h^−1^. The rates in mmol C ml^−1^ h^−1^ we quantified for our strain with Fe(II) were in the lower range of this. Autotrophic CO_2_ fixation in *Hydrogenovibrio* is operated by the Calvin-Benson-Bassham cycle with RubisCO as the key carboxylating enzyme [14, 33]. The specific RubisCO activity of *Hydrogenovibrio* strain 104 grown under H_2_:CO_2_:O_2_ (79:20:1) atmosphere was 41.5 ± 7 nmol RuBP per min and mg of protein crude extracts in the exponential growth phase which is 3.0 to 9.9 times lower then what has been measured for other *Hydrogenovibrio* isolates (Table 2).

**Table 2:** CO_2_ fixation rates for strain 104 and specific RubisCO activities for strain 104 and other *Hydrogenovibrio* species.

**CO**_**2**_ **fixation rates**	mmol C-fixation ml^−1^ h^−1^	fmol C-fixation cell^−1^ h^−1^	
S_2_O_3_^2−^ oxidation strain 104	1.26x10^−4^ ± 9.31x10^−6^	23.30 ± 1.72	
H_2_ oxidation strain 104	5.16x10^−6^ ± 3.27x10^−7^	0.29 ± 0.019	
Fe(II) oxidation strain 104	7.77x10^−7^ ± 7.52x10^−7^	0.09 ± 0.082	
**RubisCO activities**	**nmol RuBP min^−1^ mg^−1^**	**reference**	**method**
*H. crunogenus* TH-55	126 ± 8	[28]	HPLC
*H. crunogenus* XCL-2	410 ± 20	[37]	^14^C incorporation
*H. thermophilus* I78	222 ± 40	[76]	HPLC
*Hydrogenovibrio* 104	41 ± 7	this study	HPLC

This significant difference in RubisCO activities might reflect that RubisCO enzymes of strain 104 expressed under the given cultivation conditions actually have comparatively poor catalytic properties. However, the apparently reduced RubisCO activity could just as well be caused by methodological biases in the quantification of RubisCO activities (e.g., ^14^C incorporation versus HPLC assay, see Table 2). The transcriptomic data, though, suggest that genes associated with the carboxysome operon are downregulated in H_2_-treated cultures, which could also explain the rather low RubisCO activity (more information follow in section below).

### Transcriptomic analyses under different growth conditions

Transcriptomic analyses were performed for strain 104 under the three growth conditions, namely with Fe(II), H_2_, and S_2_0_3_^2−^ and revealed significant differences in the expression patterns (Fig. S6). Whereas over 500 genes are differentially expressed (FDR ≤ 0.05 and |log_2_FC| ≥ 1) when comparing S_2_O_3_^2−^ against H_2_, about twice as many are observed in comparisons including FeCl_2_ (Table S4). Here we present the most important findings regarding Fe(II), H_2_, and S_2_O_3_^2−^ oxidation and C-fixation. A more detailed description of the results from the transcriptome analysis can be found in the SI.

### Transcriptomic shifts of genes related to energy metabolism.

**Fig. 4:**
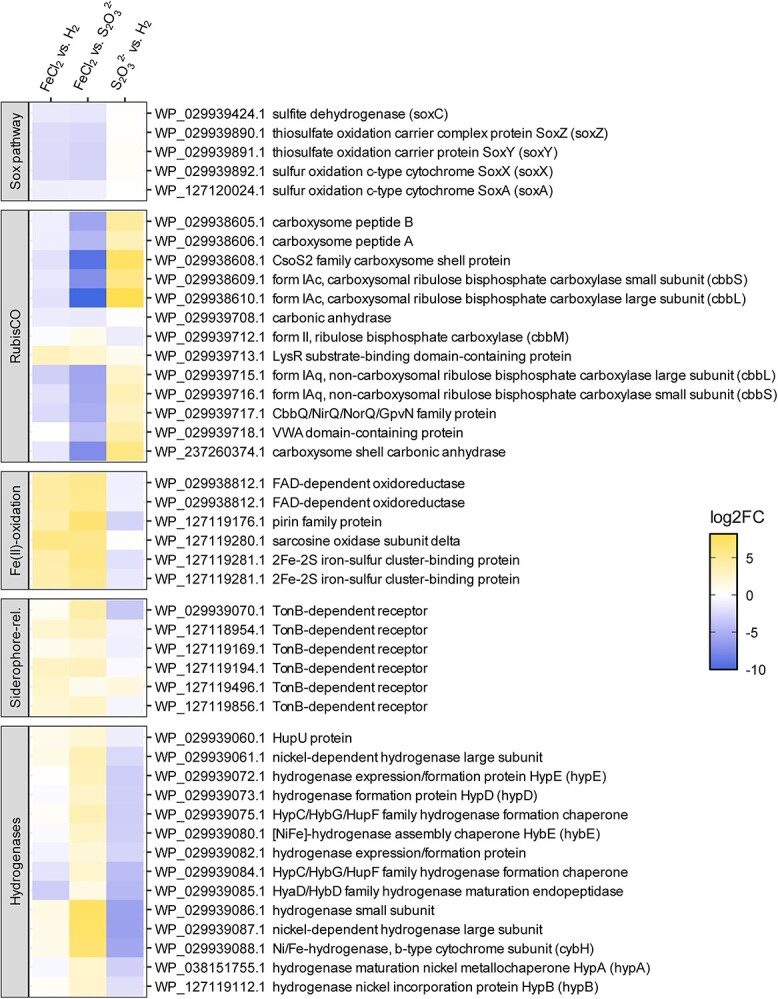
Transcription of selected genes related to metabolic sulfur, iron, or hydrogen oxidation in *Hydrogenovibrio*. Each gene is differentially regulated in at least one of the three comparisons. An extended heatmap can be found in Fig. S4.

There are only two known genes involved in neutrophilic microaerophilic Fe(II) oxidation (*cyc2* and *mtoAB*) [30]. Neither is included in the reference annotation. A number of genes encoding redox proteins (oxidoreductases) were induced with Fe(II) versus S_2_O_3_^2−^ that likely relate to electron transport from iron to oxygen, most prominently genes of the sarcosine oxidase subunit delta (Fig. 4). A genetic association with formyltetrahydrofolate deformylase (*purU*) and a high induction of the system points to a highly important interrelation of C1-metabolism and sarcosine oxidase. The physiological function of this sarcosine oxidase and the importance of it specifically during growth with Fe(II) would be an interesting aspect for future research. Genes involved in Fe transport and storage were upregulated during growth on Fe(II), most prominently a TonB-dependent receptor. In Gram-negative bacteria the TonB-dependent receptor mediates the transport of siderophores into the periplasm (together with *ExbB* and *ExbD*, which are also in the transcriptome but were not significantly upregulated with Fe(II) (Fig. S4) [34–36]. Moreover, a Pirin family protein, 2Fe-2S iron–S cluster binding protein, and some hypothetical proteins were upregulated. As these are specifically induced under Fe(II)-oxidizing conditions, these enzymes likely are somehow involved in Fe oxidation or the subsequent electron transport pathway in this organism.

Hydrogenases (H_2_-converting enzymes) were detected on the genome of *Hydrogenovibrio* already in 2006 [37], but H_2_ oxidation was experimentally verified only ~10 years later [5, 38]. *Hydrogenovibrio* species have been shown to possess [NiFe]-hydrogenases of group 1 (*hyaAB*) and group 2b (*hupUV*) [5, 14, 38]. Hydrogenase-related genes were upregulated in cells grown on H_2_ relative to those on S_2_O_3_^2−^ (Fig. 4). Unexpectedly, for cells grown with Fe(II), some hydrogenase-related genes were upregulated relative to those from H_2_ amended incubations.

*Hydrogenovibrio* species rely on the Sox enzyme system and the sulfide:quinone reductase (Sqr) for S_2_O_3_^2−^ oxidation [14, 37]. Strain 104 has an incomplete Sox pathway, *soxB*, *soxD,* and *sqr* genes are missing. The incomplete pathway results in the intra- or extracellular accumulation of ZVS [39, 40]. Genes *soxZ*, *soxC*, *soxY,* and *soxX* were upregulated in the S_2_O_3_^2−^ vs. H_2_ comparison, whereas *soxA* was slightly downregulated (Fig. 4). When the strain was grown with Fe(II) and H_2_, generally *sox* genes were downregulated and genes needed for assimilatory sulfate reduction (*cysD* and *cysN*, APS reductase, *cysI, cysG*) were upregulated, showing that the strain used sulfate, the sole S-source in these media, for assimilation.

### Transcriptomic shifts of genes related to autotrophic CO_2_-fixation

Transcriptomic data indicate that strain 104 features multiple RubisCO enzymes, which may allow to facilitate CO_2_ fixation at a variety of CO_2_ and O_2_ exposures, as has been shown for other *Hydrogenovibrio* strains before [41]. A total of three RubisCOs types were found to be transcribed in *Hydrogenovibrio* strain 104, namely (i) a carboxysomal form IA (IAc), (ii) a non-carboxysomal form IA (IAq), and (iii) a form II RubisCO. Both, form IAc and IAq RubisCO structural genes (*cbbLS*) were markedly upregulated when *Hydrogenovibrio* strain 104 grew with S_2_O_3_^2−^(Fig. 4), which coincides with the enhanced growth observed under this treatment (Fig. 3). RubisCO form II is compared to RubisCO form I the faster catalyst, which, however, comes at the expense of a lower specificity towards CO_2_ and results in lower tolerances to oxygen [42]. Using *Hydrogenovibrio marinus* it was demonstrated that the three RubisCO operons are regulated in response to CO_2_ concentration, with elevated CO_2_ levels (<2%) promoting CbbM synthesis [43]. The down regulation of the form II RubisCO structural gene *cbbM* in the S_2_O_3_^2−^ treatment, performed under fully oxic conditions and atmospheric CO_2_ levels is therefore plausible, as is its significant upregulation in the low-O_2_ but high-CO_2_ treatments, i.e., cultures grown with H_2_ and FeCl_2_ in a 20:80 CO_2_:N_2_ atmosphere with 1% O_2_. However, the non-carboxysomal form IAq is significantly downregulated when comparing H_2_ and FeCl_2_ treatments, although the starting O_2_ and CO_2_ levels are identical in both experiments, suggesting that the O_2_ and CO_2_ levels are not the only factor determining the expression of RubisCO, but that available electron donors are directly or indirectly also of relevance.

The carboxysomal RubisCO form IAc of strain 104 is arranged in a carboxysome operon together with the carboxysome shell proteins and a carboxysomal carbonic anhydrase. Thus, *Hydrogenovibrio* strain 104, like other *Hydrogenovibrio* species, possesses a carbon concentrating mechanism (CCM) that facilitates the active uptake of dissolved inorganic carbon to generate up to 100-fold increased intracellular versus extracellular concentrations [44, 45]. Therefore it is reasonable that the genes related to CCM (carbonic anhydrases and carboxysomes) were highly upregulated when strain 104 grew with S_2_O_3_^2−^ under atmospheric i.e., comparatively low CO_2_ concentrations, versus FeCl_2_- and H_2_-treated cultures growing under high CO_2_ levels, i.e., with a CO_2_:N_2_ headspace of 20:80 (Fig. 4). Additional information on RubisCO associated genes (*cbbQO* and *lysR*) can be found in the SI.

## Discussion

We describe three newly isolated strains of *Hydrogenovibrio* from hydrothermal vents of the IR, able to grow autotrophically by Fe(II), H_2_, and S_2_O_3_^2−^ oxidation. This study highlights the metabolic flexibility of *Hydrogenovibrio* providing a competitive advantage over other organisms to thrive in various vent environments.

All fluids from which the enrichments originate contained H_2_S (<10–60 μM) and Fe(II) (1.3–45 μM) [17]. High H_2_ concentrations have been measured in fluids of the Kairei vent field, located on the CIR, and previously H_2_ oxidizers have been isolated or enriched from the IR [14, 17, 46]. Given that in the environment they are exposed to various electron donors, it is beneficial if microorganisms can switch between different metabolisms or simultaneously use them. On the ZVI plates that were used for isolation of the strains, Fe(II) and H_2_ is available simultaneously as H_2_ is produced from the reduction of H_2_O by ZVI during the hydrogen evolution reaction (HER) [47], for more information see SI. The production of H_2_ per ZVI oxidized during HER is quite significant with an overall stoichiometry between 1:1 to 4:3 [47]. That the strains can also grow on FeCl_2_ and in gradient tubes with FeS in the bottom plug shows that they do not depend on H_2_ for growth but can grow autotrophically on Fe(II) alone. It is yet to be investigated which electron donor they prefer or if they can make use of them simultaneously, as it was shown previously for the Fe(II)- and H_2_-oxidizing *Ghiorsea bivora* [48], which had a growth benefit when both electron donors were present simultaneously. When strain 104 was growing on FeCl_2_ we found that hydrogenases were amongst the genes with the highest log2-fold change compared to cells grown on S_2_O_3_^2−^. This is an indication that the strain may be using both metabolisms simultaneously in the environment. ZVI plates, which were used for isolation, would provide an advantage for organisms being able to use H_2_ and Fe(II) simultaneously and therefore likely specifically select for organisms with this ability.

Most microaerophilic Fe(II)-oxidizers are described to produce twisted stalks or sheaths as characteristic Fe(III)-mineral structures [49–51]. This is different for our strains, they produce “coral-like” structures consisting of bulbous Fe(III)-minerals with ~1–6 μm in diameter (Fig. 1). These structures appear very different from Fe(III) that precipitated in abiotic controls and are similar to what is reported for *Ghiorsea bivora* [48], a Zetaproteobacterium that is also capable of Fe(II) and H_2_ oxidation, and *Hydrogenovibrio* sp. SC-1, the only other known *Hydrogenovibrio* capable of Fe(II) oxidation [15]. For *Ghiorsea* it was shown that it produces soluble exometabolites. These exometabolites are responsible for the Fe(III)-mineral formation outside the cell, that is different from abiotic mineral formation [52]. We could not find any known genes for Fe(II) oxidation in the transcriptome, therefore we conclude that, as previously suggested for SC-1 [15], the organism uses another Fe(II) oxidation pathway as indicated by Fe(III)-minerals being different from those generated by other Fe(II)-oxidizers. The genome of SC-1 was also sequenced and no known genes for Fe oxidation can be found in the genome [53].

When the strain 104 was grown on FeCl_2_, hydrogenases were upregulated compared to S_2_O_3_ [2]. Five of thirteen observed hydrogenase-related genes were significantly higher expressed with FeCl_2_ than in H_2_, whereas two were significantly less expressed (Fig. 4). However, the latter two are other hydrogenases than the ones important for H_2_ oxidation as seen from the comparison of H_2_ and S_2_O_3_^2−^, as they are also downregulated in this comparison.

It could be that hydrogenases are upregulated with FeCl_2_ as the strain potentially uses Fe(II) and H_2_ simultaneously. However, under the conditions that the Fe(II) culture was grown for the transcriptomic analyses, there is no H_2_ available. Another reason for the upregulation could be that hydrogenases themselves are somehow involved in Fe(II) oxidation. This hypothesis would require further research though. Another interesting aspect is the upregulation of TonB in the culture grown on Fe(II). One possibility for the upregulation of genes related to siderophores in our strain could be that they are involved in the formation of the Fe(III) mineral structures, similar to the soluble exometabolites that are involved in Fe(III) mineral formation in *Ghiorsea* [52]. Siderophores have been found to be abundant and diverse in hydrothermal plumes [54]. They are important in keeping Fe in solution and thereby impact cycling and transport of iron in the ocean [55]. Whereas TonB can also be involved in the transport of carbohydrates [56], especially in venting regions where carbohydrates are abundant, such as the Guaymas Basin [57]. A study that investigated microbial genes involved in Fe uptake in hydrothermal plumes in the Guaymas Basin found that TonB was amongst the most abundant genes in the whole plume metatranscriptome [58] and the authors link that to Fe transport. That our strain has the potential to be involved in siderophore production/export highlights the environmental importance that Fe-oxidizing microbes such as *Hydrogenovibrio* might have.

Thermodynamically, thiosulfate is with −762 kJ/mol S_2_O_3_^2−^ oxidized the most favorable of the investigated electron donors [59]. Hydrogen yields −237 kJ/mol H_2_ [60] and microaerophilic Fe(II) oxidation only about −90 kJ/ mol Fe oxidized [61]. Results from the ^14^C-HCO_3_^−^ incorporation experiments show that the strain can grow autotrophically with all three tested electron donors. C-fixation rates with S_2_O_3_^2−^ were much higher compared to H_2_ and Fe(II), consistent with much higher cell growth and the high upregulation of RubisCO with S_2_O_3_^2−^. Based on our measurements for C-fixation and concurrent substrate utilization, for each mole of C-fixed ca. 16, 23, and 35 moles of substrate had to be oxidized for S_2_O_3_^2−^, H_2_, and Fe(II), respectively. The ratios of substrate oxidized per C-fixed in other autotrophic organisms range from 2:1 to 5:1 for aerobic sulfide oxidizers [62–65], 10:1 for ammonium oxidizers [66, 67], and 25:1 to 80:1 for nitrite oxidizers [68]. Considering that 4 mol electrons are needed for each mol of C-fixation, this means that ~7, 9, and 11% of the electrons transferred from the substrate went into C-fixation (for C-reduction) and the remaining electrons were used for energy generation. This agrees relatively well with previous reports that ~10–20% of the electrons are used for biomass synthesis [63, 69–71]. The values for oxidized Fe(II) per fixed CO_2_ that we found for Fe(II)-oxidizing conditions are slightly lower than what was estimated previously for other neutrophilic microaerophilic Fe(II) oxidizers, for which values of 43 to 70 mol oxidized Fe(II) per mol of fixed CO_2_ were suggested [72, 73]. The rates of CO_2_-fixation were much higher for S_2_O_3_^2−^compared to FeCl_2_ and H_2_, likewise the increase in cell numbers was much faster. It will have to be elucidated in the future, which is the favored electron donor in the environment where the strains were isolated from.

In the environmental samples used for isolation of our new strains, we found that *Hydrogenovibrio/Thiomicrospira* had a relative abundance of up to 6% [17]. For the fluid from which strain 104 was isolated we counted 3.44 × 10^6^ cells ml^−1^_._ This means that we potentially have up to 2.064 × 10^5^*Hydrogenovibrio* cells per ml of hydrothermal fluid. Assuming that all these cells consume either Fe(II), H_2_, or S_2_O_3_^2−^ at the rate measured for the isolates, we could find rates of C-fixation of 1.75 × 10^−8^, 6.04 × 10^−8^, and 4.8 × 10^−6^ mmol C ml^−1^ h^−1^, respectively. Unfortunately, we do not have flow rates for the vents from which the samples originate. Based on literature, we assume rates for low-temperature diffuse vents between 116–17 580 l h^−1^ [74, 75]. Based on these numbers and the rates we measured with the cultures, between 71–10 777, 159–24 109, and 6282–952 183 μmol Fe(II), H_2_, and S_2_O_3_^2−^ could become oxidized per vent-site per hour, respectively. For C-fixation we calculated that during Fe(II), H_2_, and S_2_O_3_^2−^ oxidation by *Hydrogenovibrio* between 2–308, 7–1061, and 557–84 420 μmol C could be fixed per vent site and hour, respectively. These rates are potential rates that can be reached by a pure culture under ideal conditions. Thus, they are likely overestimates of what can be reached in the environment and many assumptions go into this calculation. The details of the calculation can be found in the SI (Table S5). Comparisons of the cell-specific ^14^C fixation rates from our strain (Table 2) and rates determined in hydrothermal fluids, which were in the range of 0.0001–0.1 fmol cell^−1^ h^−1^ [31, 32], show that our rates are high. However, in another study ^14^C fixation rates with Fe(II) in hydrothermal fluids have been in the same range [6] (4 × 10^−7^ to 20 × 10^−7^ mmol C ml^−1^ h^-1^, Table 2). Even though our estimates are rough, the calculations demonstrate that *Hydrogenovibrio* potentially substantially contributes to C, S, Fe, and H_2_ cycling at these vents.

The metabolic versatility of the isolated strains highlight that *Hydrogenovibrio* species do not only play an important role in S and carbon cycling, but are also important in H_2_ and iron cycling. Because no known genes for Fe(II) oxidation were found, we conclude that the strain uses a yet unknown pathway for Fe(II) oxidation. The metabolic versatility makes these strains good candidates for studying new Fe oxidation genes and mechanisms.

## Supplementary Material

Supplements_HydrogenovibrioINDEX2019_2024-09-17_wrae173

## Data Availability

Sequence data for the transcriptomes reported in this publication have been submitted to the European Nucleotide Archive. They are publicly available under accession PRJEB71696. The 16S rRNA gene sequences of all three strains were deposited under GenBank accession numbers PP083208, PP083209, and PP083210.
