# Supplementary material for: Oxidation of sulfur, hydrogen, and iron by metabolically versatile Hydrogenovibrio from deep sea hydrothermal vents
Source: ISME J. 2024 Sep 14;18(1):wrae173. doi: 10.1093/ismejo/wrae173 (PMC11439405; doi:10.1093/ismejo/wrae173)
Supplement: Supplements_HydrogenovibrioINDEX2019_2024-09-17_wrae173 [file supplements_hydrogenovibrioindex2019_2024-09-17_wrae173.docx]

Oxidation of sulfur, hydrogen, and iron by metabolically versatile Hydrogenovibrio from deep sea hydrothermal vents

Laufer-Meiser K.^1*^, Alawi M.^2^, Böhnke S.^1^, Solterbeck C.H.^3^, Schloesser J.^3^, Schippers A.^4^, Dirksen P^2^, Brüser T.^5^, Henkel S.^6^, Fuss J.^7^ and Perner M.^1^*

^1^Marine Geosystems, GEOMAR Helmholtz Centre for Ocean Research Kiel, Kiel Germany

^2^Bioinformatics Core, University Medical Center Hamburg-Eppendorf, Hamburg, Germany

^3^Institute for Materials and Surfaces, Kiel University of Applied Sciences, Kiel, Germany

^4^Federal Institute for Geosciences and Natural Resources (BGR), Hannover, Germany

^5^Institute of Microbiology, Leibniz Universität Hannover, Hannover, Germany

^6^Alfred Wegener Institute Helmholtz Centre for Polar and Marine Research, Bremerhaven, Germany

^7^Institute of Clinical Molecular Biology Kiel University, Kiel, Germany

*corresponding authors: klaufer@geomar.de and mperner@geomar.de

Supplementary information

**Supplementary Material and Methods**

**Enrichment and isolation**

ZVI (zero valent iron) plates were used for the initial enrichment. The plates were prepared with 6 cm diameter petri dishes to which sterile ZVI powder (200 mesh; metal basis; Alfa Aesar, Ward Hill, MA) was added, so that the bottom of the plate was thinly covered with the powder. Afterwards, 6 ml of ASW (Table S3) was added. For initial enrichment, 1 ml of the hydrothermal fluid was added to the plate. The plates were incubated under microoxic conditions (O_2_ = 6-10% of atmospheric O_2_) that are created with gas packs (BD GasPack EZ Campy; Becton, Dickinson and Co., NJ) inside anoxic jars (Merck, Darmstadt, Germany). On the surface of the liquid medium a shiny film developed (Figure S1), which contained Fe minerals to which microbial cells were attached (Figure 1). Material from the shiny film was transferred on the ZVI plates by picking up parts of the film and some of the liquid medium with a sterile Pasteur pipette. For dilution to extinction, the first ZVI plate was inoculated as described above, then the plate was gently mixed and 10-fold dilutions were prepared by pipetting 600 µl into the next ZVI plate. This was done down to a dilution of 10^-8^. After several transfers the culture appeared homogeneous and to transfer the culture into MJ medium to test for growth on H_2_ some of the liquid medium from the ZVI plates was taken up with a syringe and injected into a Hungate tube containing MJ medium.

Through the reduction of water coupled to the oxidation of ZVI, H_2_ is generated by the hydrogen evolution reaction (HER) by the following reaction1 (Equation 1):

$3 {Fe}^{0}+4 H_{2}O\to{Fe}_{3}O_{4}+4H_{2}$ Eq. 1

**Quantification of RubisCO enzyme activity**

**Preparation of crude extracts.** *Hydrogenovibrio* strain 104 cultures were grown in 100 ml serum vials with 50 ml MJ medium and a H_2_/CO_2_/O_2_ (79:20:1) headspace. A culture batch of 1l was harvested at the end of the exponential growth phase by centrifugation (10,000 x g, 30 min, 4°C). Cell pellets were washed twice with pre-chilled RubisCO assay buffer [100 mM Tris‑HCl (pH 7.8), 10 mM MgCl_2_, 1 mM EDTA, 25 mM NaHCO_3_, and 1 mM DTT] and resuspended in 2 ml of the same buffer. Cells were mechanically disrupted using the French pressure cell press (Thermo Spectronic) and cell debris were subsequently removed by centrifugation (12 800 x g, 20 min, 4°C). Protein quantification was done after Bradford and Williams (1976) using 5x Roti®Quant Bradford-solution (Carl Roth GmbH + Co. KG, Karlsruhe, Germany).

**RubisCO activity assay.** The RubisCO activity assay was performed at 25 °C in RubisCO assay buffer with a total of 0.2 mg µl^-1^ protein crude extract. 10 mM ribulose-1,5-bisphosphate (RuBP) was added to start the reaction. Subsamples were taken and heat inactivated (95°C, 3 min) at 0, 10, 30 and 120 minutes. Denatured proteins were removed by centrifugation (16,100 x g, 20 min, 4 °C) and clear supernatants were used to quantify the consumption of RuBP and the production of 3-phosphoglycerate (3-PGA) on a LaChrom Elite® HPLC system (Hitachi, Tokio, Japan) with a Lichrospher® 100 RP 18e column (VWR International GmbH, Darmstadt, Germany). Separation was done at 22°C via ion pair chromatography with 0.05 M Tetrabutylammonium hydrogensulfate (Merck, Darmstadt, Germany) in isocratic runs with 10 % acetonitrile (v/v) as eluent and a flow rate of 0.6 ml per minute. Detection was carried out in a DAD-detector set to 207 nm. A protein free reference sample with 10 mM RuBP was used as an internal standard.

**Supplementary Results and Discussion**

**Isolation of three *Hydrogenovibrio* strains from IR vents**

After >10 transfers with dilution to extinction series on ZVI plates, cells appeared uniform and most cells were attached to Fe-minerals. The cultures formed a floating shiny film on surface of ZVI plates (Figure S1). Under the microscope, Fe-minerals are recognized as bulbous structures that stick together, forming “coral-like” forms (Figure 1). The structures appeared red under the light microscope, suggesting that they were composed of Fe(III).

**Growth of three *Hydrogenovibrio* strains on Fe(II), H_2_ and S_2_O_3_^2-^ and quantification of oxidation rates**

**Growth on various Fe(II) substrates, H_2_ and S_2_O_3_^2-^.** After isolation of the cultures 040, 083, and 104 on ZVI plates, they were tested for their ability to grow on various forms of Fe(II), H_2_ and different reduced sulfur compounds. In FeS gradient tubes, growth of the cultures was obvious from the oxidation ring, that was more narrow and confined in the inoculated gradient tubes compared to the controls (Figure S2). Microscopic observation of a sample taken from the growth band confirmed the presence of cells that were attached to Fe-minerals. On FeCl_2_, growth was confirmed by microscopy of the cultures (Figure S3), where cells attached to Fe-minerals were also found. The strains were also able to grow on T-ASW plates forming smooth and round white colonies causing a color change of the agar from red to yellow. Further growth experiments were carried out with H_2_S as electron donor and nitrate as electron acceptor, but no growth was observed under these conditions (Table 1).

**Microbial Fe(II) oxidation rates.** In incubations to which FeCl_2_ was repeatedly added, Fe(II) concentrations were kept considerably lower in the inoculated incubations compared to the uninoculated controls (Figure 3). This difference showed that there was biotic Fe(II) oxidation in addition to abiotic Fe(II) oxidation in the abiotic controls. Fe(II) oxidation rates were calculated from the difference between the biotic and abiotic incubations.

**CO_2_ fixation rates with Fe(II), H_2_ or S_2_O_3_^2-^ as inorganic electron donors**

C-fixation per cell and hour for strain 104 was about 130 and 270 times higher with thiosulfate compared to H_2_ and Fe(II), respectively (Table 2)

**Transcriptomic analyses under different growth conditions**

**Choice of the reference genome.** We chose the reference genome of strain S5 because of its high similarity to our strain based on 16S rRNA (100%). We also did the same analysis with strain JR-2, which is also highly similar to our strain based on the 16S rRNA gene (100%). However, the genome of JR-2 was lacking hydrogenases this is why we decided for the genome of S5.

The most up- and downregulated genes, based on log2-fold change (L2FC) are shown in Table S1 and S2. Figure S4 shows a heatmap of the L2FC of the genes mentioned in the manuscripts and the supplemental information.

**Transcriptomic shifts related to Fe metabolism**

In the transcriptome of the cells grown on Fe(II), the annotated 2Fe-2S iron-sulfur cluster-binding protein, and the FAD-dependent oxidoreductase in fact correspond to subunits alpha and beta of sarcosine oxidase, and all these are encoded in an operon together with the upregulated formyltetrahydrofolate deformylase gene *purU*, which is in that case unlikely to be involved in purine biosynthesis but more likely forms a complex with sarcosine oxidase. Sarcosine oxidases bind tetrahydrofolate, which binds the released formaldehyde and therefore usually releases 5,10-methylenetetrahydrofolate that is used in the C1-metabolism^2–4^. The genetic association with formyltetrahydrofolate deformylase with sarcosine oxidase and the high induction of the system point to a highly important interrelation of C1-metabolism and sarcosine oxidase in this organism under these growth conditions. Subunit gamma is absent, and instead a hypothetical protein is translationally coupled to the alpha subunit, further substantiating an unusual variation of the tetrameric type of sarcosine oxidase in this organism.

**Transcriptomic shifts related to S_2_O_3_^2-^ metabolism**

The S_2_O_3_^2-^-oxidizing bacteria showed strongest downregulation of the genes encoding sulfate assimilation-related enzymes, including sulfate activation (CysD and CysN), APS reductase, assimilatory sulfite reductase (*cysI*) and the siroheme synthase (*cysG*). Siroheme is the redox cofactor of the assimilatory sulfite reductase. The down-regulation of these enzymes indicates that sulfur assimilation with sulfate as sulfur source is not required and therefore downregulated when the strain is growing on thiosulfate. Most likely, the sulfane sulfur from thiosulfate can be directly used as sulfur source for incorporation into organic compounds. Rhodaneses could in principle aid in the transfer of sulfane sulfur to organic molecules. Rhodaneses are defined as enzymes that are able to transfer the sulfane sulfur of thiosulfate to the artificial acceptor cyanide, and physiological functions that have been found for rhodaneses or rhodanese-domain-containing proteins are diverse and often unknown. In *Escherichia coli*, a rhodanese (PspE) has recently been shown to be responsible for the acquisition of thiosulfate sulfane sulfur for cellular sulfane sulfur^5^. Interestingly, while the expression of genes encoding five rhodanese-domain-containing proteins is not increased during growth with S_2_O_3_^2-^, the gene of one rhodanese-domain-containing protein is significantly upregulated, and it is therefore possible that this protein is responsible for the use of sulfane sulfur for sulfur assimilation in this organism. When the strain was grown with FeCl_2_ and H_2_ genes needed for assimilatory sulfate reduction were upregulated, which shows that the strain used sulfate, which was present in both media as sole S-source, for assimilation.

**Transcriptomic shifts of genes related to CO_2_-fixation.**

Similar to what has been described for other *Hydrogenovibrio crunogenus* RubisCO gene arrangements, the structural RubisCO genes of the non-carboxysomal RubisCO form IA (*cbbLS*) and the RubisCO form II (*cbbL*) are juxtaposed, separated by two LysR-type regulatory proteins encoding genes and are oriented in opposite directions (Böhnke et al 2014; Scott et al 2006). In a heterologously expressed *Hydrogenovibrio*-related non-carboxysomal RubisCO gene cluster, it has already been shown that LysR binding sites upstream of *cbbL* and *cbbM* allow binding of these LysR transcriptional regulators and that *cbbM* is up-regulated if its neighboring *lysR2* is transcribed (Böhnke and Perner, 2017). Therefore, it is consistent that *lysR*, which is located right next to the RubisCO form II encoding gene, is upregulated together with *cbbM*, i.e. when H_2_ and FeCl_2_ are available. The second LysR regulator upstream of the *cbbL* gene, is not up- or down-regulated in any of the treatments, and thus appears to be constitutively expressed independently of the prevailing O_2_ or CO_2_ concentrations or the available electron acceptor. The structural RubisCO genes of the non carboxysomal form IA and the form II RubisCO of strain 104 are each followed by RubisCO activase encoding genes *cbbQ and cbbO*, forming the two clusters *cbbMQO* and *cbbLSQM*, which is a common arrangement for *Hydrogenovibrio* species (SI Figure S3 of Boehnke et al 2019). A model based on mutation experiments suggests, that the AAA+ protein CbbQ acts as motor and CbbO as a substrate adaptor that binds RubisCO via a von Willebrand factor A domain^6^. For *Acidithiobacillus ferrooxidans*, which has a similar RubisCO gene cluster arrangement as strain 104, it was shown that the CbbQO activase system is specific for the RubisCO enzyme encoded in the same gene cluster^7^. However, this specificity could not be experimentally verified for a heterologously expressed RubisCO gene cluster from an uncultured *Hydrogenovibrio crunogenus*-related species^8^. The expression patterns of the *cbbQO* and the *cbbLS* show a very similar response in all three treatments, which may indicate possible co-dependence. The activase system from the *cbbMQO* cluster, on the other hand, is not upregulated together with *cbbM*, which rather contradicts a highly connected specificity.

The gene encoding the second key enzyme of the CBB cycle, phosphoribulokinase (PRK), is constitutively transcribed in all three treatments (Figure S4).

**Other relevant transcriptomic shifts.**

Beyond carbon fixation, upregulated genes in the stronger growing S_2_O_3_^2-^-oxidizing cells also related to periplasmic disulfide bond formation (*dsrE* and *dsrB*), transport (TolC family proteins, RND transporters, chromate transporter, ABC-transporter binding proteins), biosynthesis (spermidine synthase SpeE)^9^, degradation pathways (hydrolases, heme binding and degradation) and regulation (MCP, LysR family regulator, TfoX). All of these processes reflect the higher metabolic activity during growth with thiosulfate as electron donor.

Genes related to flagella were amongst the genes that were differently regulated in the comparison of the three growth conditions (Figure S5). The figure shows that with S_2_O_3_^2-^, genes related to flagella are downregulated, while they are upregulated with H_2_ and FeCl_2_, while the highest upregulation was found with FeCl_2_, indicating that under these conditions it was beneficial for the cells to be more motile, likely to reach O_2_ which was diffusing into the medium from the surface. In case of FeCl_2_, an even higher mobility compared to H_2_ might be beneficial because of the stronger gradients that form due to the abiotic reactions between O_2_ and Fe(II).


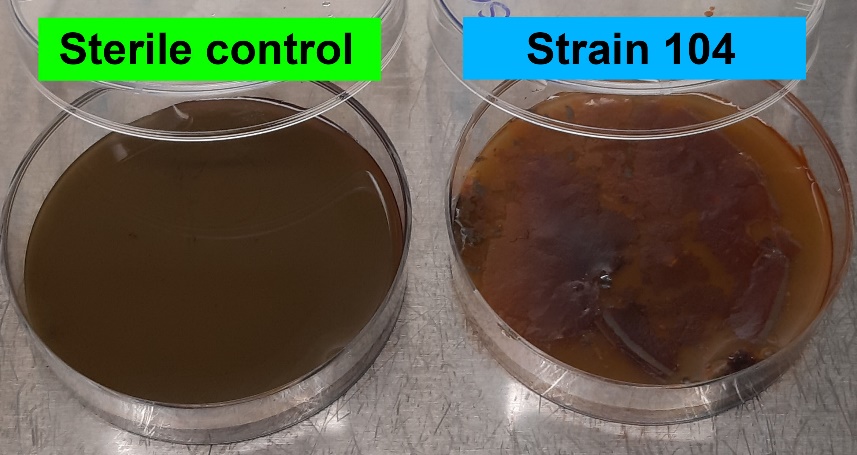


**Figure S1**: ZVI plates. On the left uninoculated control, on the right inoculated with strain 104. The shimmering film on the surface of the ASW medium can be seen.


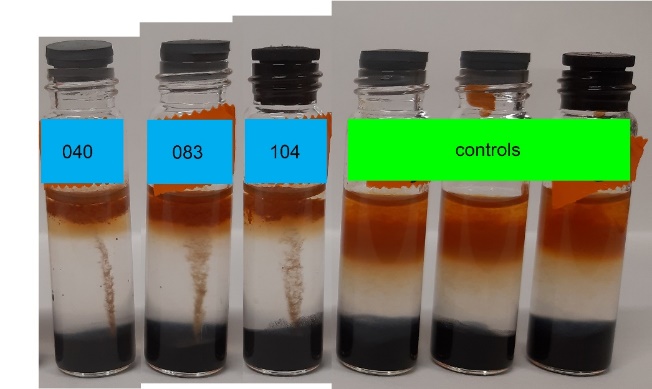


**Figure S2:** Fe-gradient tubes. The three tubes on the left were inoculated with strain 040, 083 and 104. As indication of growth, they show a narrower and more confined band of oxidized Fe compared to the three controls on the left.


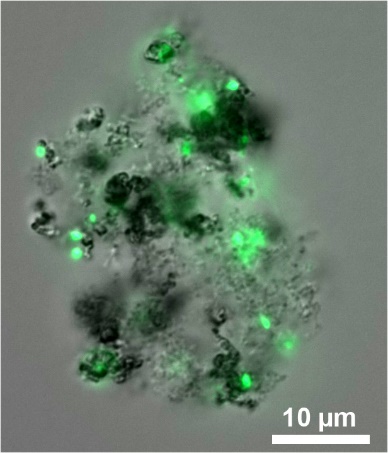


**Figure S3:** Cells with minerals grown in FeCl_2_. The cells are stained with live-death stain (Invitrogen, Waltham, MS, USA), shown is the SYTO 9 stain in green.

**
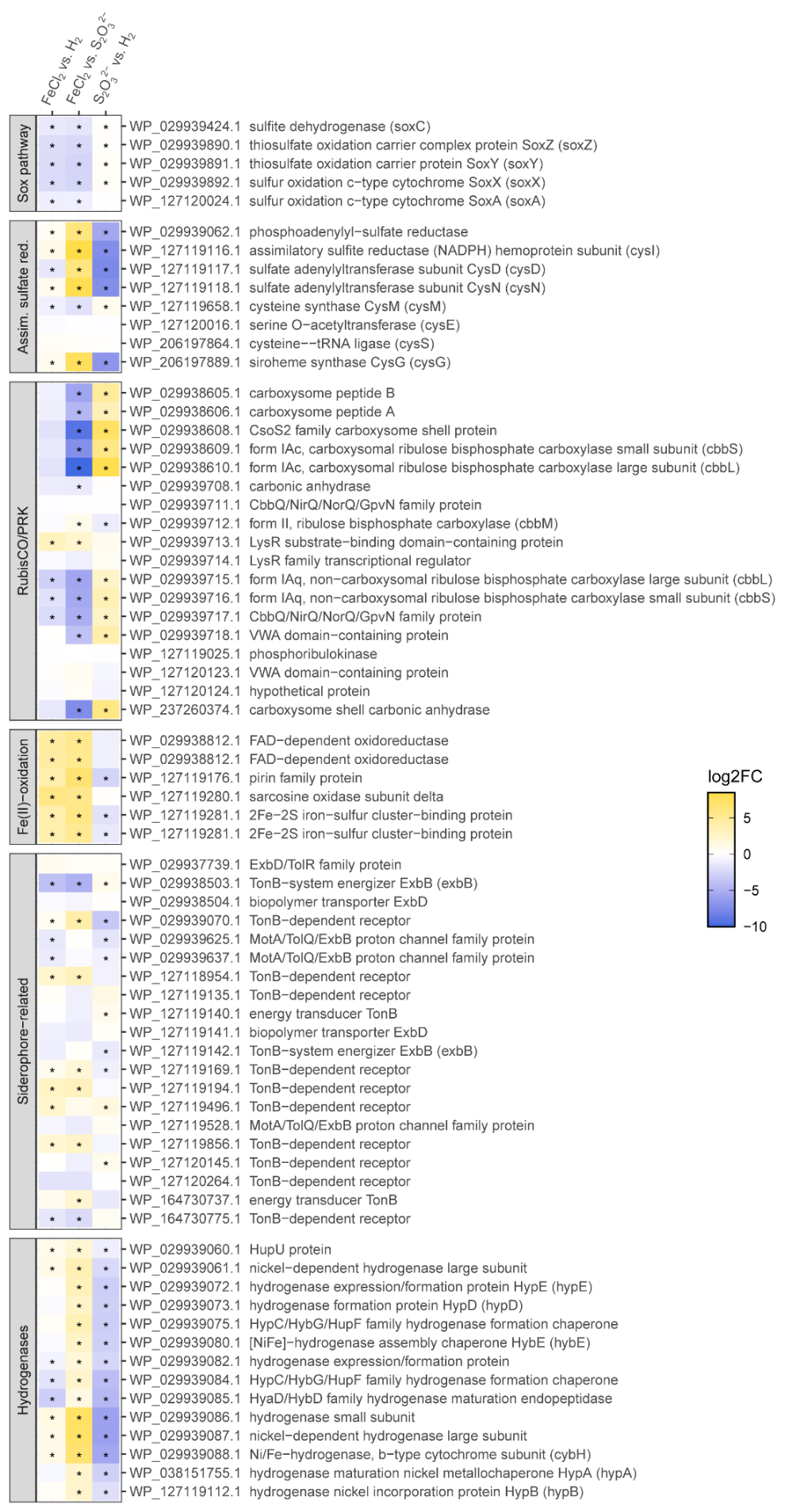
**

**Figure S4:** Transcription of selected genes related to metabolic sulfur, iron, or hydrogen oxidazion in Hydrogenovibrio. Significant comparisons are marked with *.

***
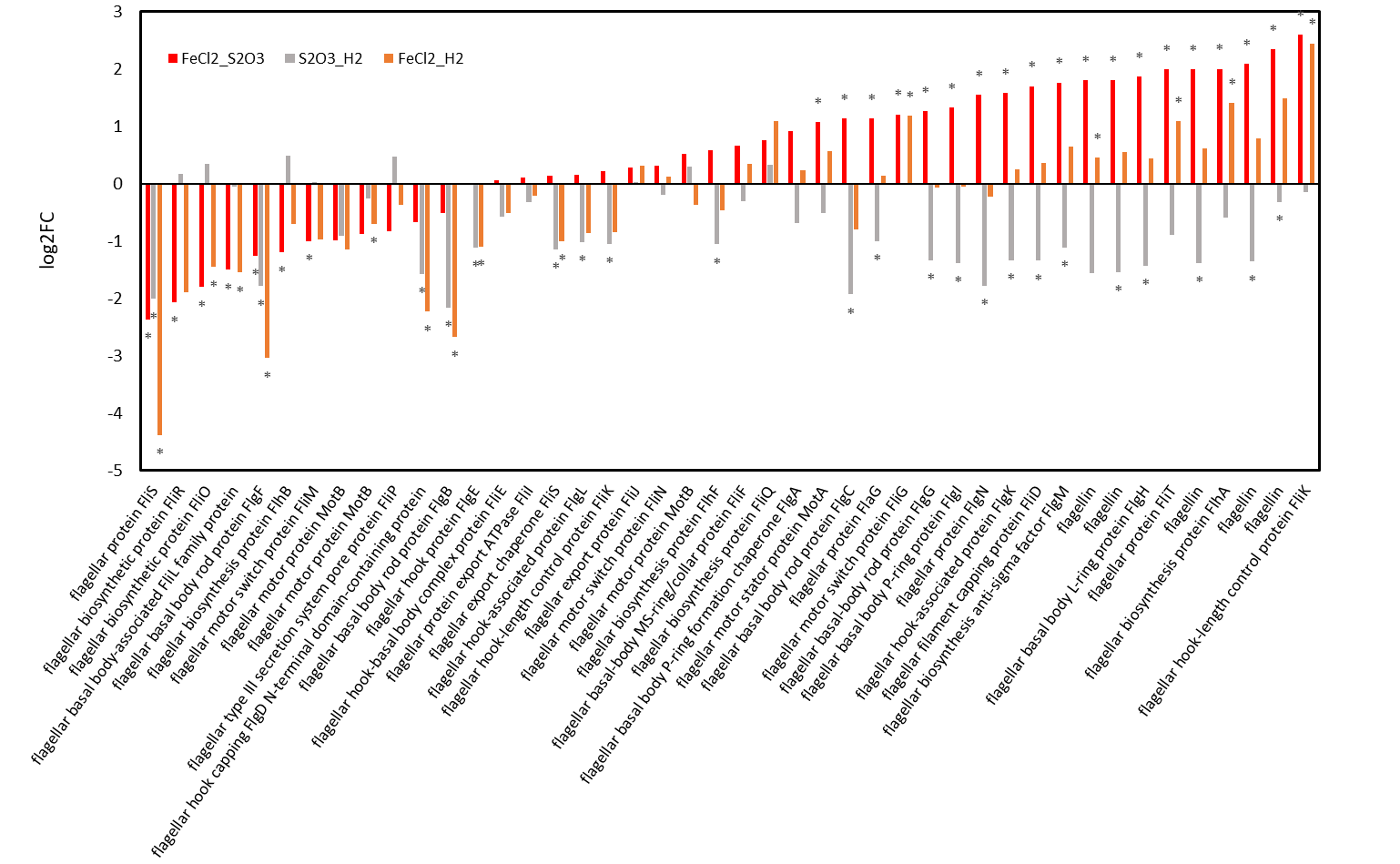
***

***Figure S5:*** *Up- and downregulation of genes related to flagella. Significant comparisons are marked with *.*


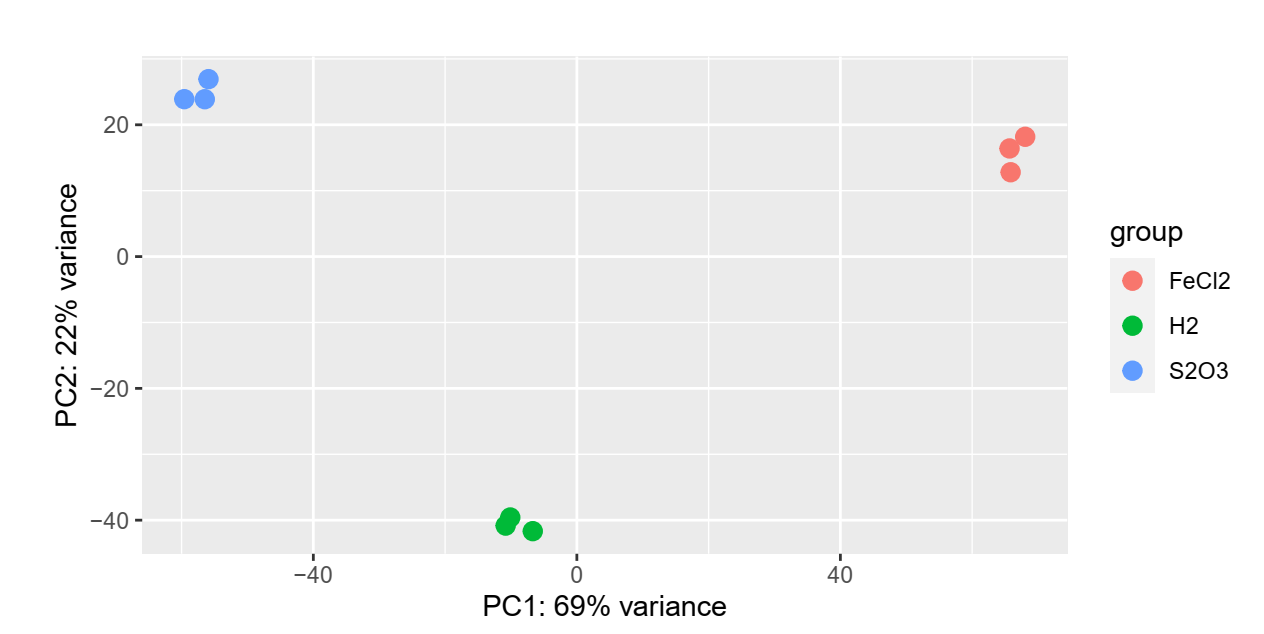


**Figure S6**: PCoA of transcriptomes grown on Fe (II), H_2_ or S_2_O_3_^2-^.

***Table S1:*** *The 25 significantly differentially expressed protein-coding genes with the highest Log_2_-fold changes (Log2FC).*

|  | **FeCl2_S2O3** | | **S2O3_H2** | | **FeCl2_H2** | |
| --- | --- | --- | --- | --- | --- | --- |
|  | Gene (protein id, gene symbol, product) | Log2FC | Gene (protein id, gene symbol, product) | Log2FC | Gene (protein id, gene symbol, product) | Log2FC |
| 1 | WP_127119116.1, cysI, assimilatory sulfite reductase (NADPH) hemoprotein subunit | 8.46 | WP_029938610.1, form I ribulose bisphosphate carboxylase large subunit | 8.18 | WP_127119280.1, sarcosine oxidase subunit delta | 5.85 |
| 2 | WP_127119118.1, cysN, sulfate adenylyltransferase subunit CysN | 8.11 | WP_029938608.1, CsoS2 family carboxysome shell protein | 7.36 | WP_127119192.1, aminotransferase class I/II-fold pyridoxal phosphate-dependent enzyme | 5.60 |
| 3 | WP_206197889.1, cysG, siroheme synthase CysG | 7.60 | WP_029938597.1, BMC domain-containing protein | 7.01 | WP_127118951.1, response regulator | 5.08 |
| 4 | WP_029939086.1, hydrogenase small subunit | 7.36 | WP_127119405.1, rdgB, RdgB/HAM1 family non-canonical purine NTP pyrophosphatase | 6.13 | WP_029938812.1, FAD-dependent oxidoreductase | 4.54 |
| 5 | WP_029939087.1, nickel-dependent hydrogenase large subunit | 7.21 | WP_029910948.1, BMC domain-containing protein | 5.96 | WP_029938812.1, FAD-dependent oxidoreductase | 4.47 |
| 6 | WP_127119115.1, assimilatory sulfite reductase (NADPH) flavoprotein subunit | 7.09 | WP_237260374.1, carboxysome shell carbonic anhydrase | 5.93 | WP_029939415.1, thiS, sulfur-carrier protein ThiS | 4.31 |
| 7 | WP_029939088.1, cybH, Ni/Fe-hydrogenase, b-type cytochrome subunit | 6.76 | WP_029938609.1, ribulose bisphosphate carboxylase small subunit | 5.89 | WP_127119952.1, hypothetical protein | 4.17 |
| 8 | WP_127119176.1, pirin family protein | 6.75 | WP_127119402.1, ferritin-like domain-containing protein | 5.73 | WP_029938813.1, purU, formyltetrahydrofolate deformylase | 4.12 |
| 9 | WP_029939037.1, FMN-dependent NADH azoreductase | 6.52 | WP_029939175.1, NADH-quinone oxidoreductase subunit L | 5.06 | WP_164730772.1, hypotehtical protein | 4.07 |
| 10 | WP_029939062.1, phosphoadenylyl-sulfate reductase | 6.24 | WP_029938603.1, BMC domain-containing protein | 4.98 | WP_127119176.1, pirin family protein | 4.04 |
| 11 | WP_127119117.1, cysD, sulfate adenylyltransferase subunit CysD | 6.17 | WP_029938604.1, BMC domain-containing protein | 4.93 | WP_127119281.1, 2Fe-2S iron-sulfur cluster-binding protein | 3.97 |
| 12 | WP_127119281.1, 2Fe-2S iron-sulfur cluster-binding protein | 5.85 | WP_127119403.1, hypothetical protein | 4.83 | WP_127119281.1, 2Fe-2S iron-sulfur cluster-binding protein | 3.88 |
| 13 | WP_127119280.1, sarcosine oxidase subunit delta | 5.62 | WP_029938605.1, carboxysome peptide B | 4.81 | WP_127118953.1, 4Fe-4S binding protein | 3.87 |
| 14 | WP_029938812.1, FAD-dependent oxidoreductase | 5.50 | WP_127119404.1, hypothetical protein | 4.76 | WP_127118942.1, DFU domain-containing protein | 3.72 |
| 15 | WP_237260355.1, SLC13 family permease | 5.43 | WP_029938601.1, 4a-hydroxytetrahydrobiopterin dehydratase | 4.40 | WP_127119043.1, YigZ family protein | 3.66 |
| 16 | WP_029938812.1, FAD-dependent oxidoreductase | 5.33 | WP_127120281.1, cytochrome b | 4.27 | WP_127119101.1, SDR family NAD(P)-dependent oxidoreductase | 3.49 |
| 17 | WP_127119280.1, sarcosine oxidase subunit delta | 5.28 | WP_127120211.1, MBL fold metallo-hydrolase | 4.24 | WP_127119280.1, sarcosine oxidase subunit delta | 3.45 |
| 18 | WP_127119281.1, 2Fe-2S iron-sulfur cluster binding protein | 5.25 | WP_029937996.1, AAA family ATPase | 4.17 | WP_127119921.1, DsrE family protein | 3.43 |
| 19 | WP_127119031.1, efflux RND transporter preiplasmic adaptor subunit | 5.14 | WP_029939718.1, VWA domain-containing protein | 4.02 | WP_127119951.1, MarR family winged helix-turn-helix transcriptional regulator | 3.40 |
| 20 | WP_127119192.1, aminotransferase class I/II-fold pyridoxal phosphate-dependent enzyme | 5.04 | WP_206197882.1, DsrE family protein | 3.89 | WP_127120299.1, FAD-dependent oxidoreductase | 3.36 |
| 21 | WP_029939838.1, (2Fe-2S)-binding protein | 4.42 | WP_127119764.1, disulfide bond formation protein B | 3.63 | WP_029939520.1, DMT family transporter | 3.33 |
| 22 | WP_127118951.1, response regulator | 4.26 | WP_029939716.1, ribulose bisphosphate carboxylase small subunit | 3.61 | WP_127119013.1, hypothetical protein | 3.29 |
| 23 | WP_127119099.1, DUF1365 domain-containing protein | 4.12 | WP_029938606.1, carboxysome peptide A | 3.52 | WP_127118922.1, hypothetical protein | 3.28 |
| 24 | WP_029939070.1, TonB-dependent receptor | 4.11 | WP_127119766.1, DUF4395 domain-containing protein | 3.37 | WP_164730708.1, hypothetical protein | 3.28 |
| 25 | WP_127118922.1, hypothetical protein | 4.08 | WP_127119330.1, eutC, ethanolamine ammonia-lyase subunit EutC | 3.25 | WP_127119330.1, eutC, ethanolamine ammonia-lyase subunit EutC | 3.25 |

**Table S2:** The 25 significantly differentially expressed protein-coding genes with the lowest Log_2_-fold changes (Log2FC).

|  | **FeCl2_S2O3** | | **S2O3_H2** | | **FeCl2_H2** | |
| --- | --- | --- | --- | --- | --- | --- |
|  | Gene (protein id, gene symbol, product) | Log2FC | Gene (protein id, gene symbol, product) | Log2FC | Gene (protein id, gene symbol, product) | Log2FC |
| 1 | WP_029938610.1, form I ribulose bisphosphate carboxylase large subunit | -10.00 | WP_127119117.1, cysD, sulfate adenylyltransferase subunit CysD | -7.72 | WP_029938519.1, DUF492 domain-containing protein | -5.40 |
| 2 | WP_029938608.1, CsoS2 family carboxysome shell protein | -9.25 | WP_127119116.1, cysI, assimilatory sulfite reductase (NADPH) hemoprotein subunit | -7.31 | WP_029938870.1, rpmG, 50S ribosomal protein L33 | -5.09 |
| 3 | WP_029938603.1, BMC domain-containing protein | -7.88 | WP_127119118.1, cysN, sulfate adenylyltransferase subunit CysN | -7.29 | WP_127120115.1, outer membrane protein transport protein | -4.92 |
| 4 | WP_029910948.1, BMC domain-containing protein | -7.53 | WP_206197889.1, cysG, siroheme synthase CysG | -6.77 | WP_127119275.1, permease | -4.66 |
| 5 | WP_237260374.1, carboxysome shell carbonic anhydrase | -7.38 | WP_029939087.1, nickel-dependent hydrogenase large subunit | -6.06 | WP_029938847.1, hypothetical protein | -4.59 |
| 6 | WP_127119403.1, hypothetical protein | -7.28 | WP_029939086.1, hydrogenase small subunit | -5.95 | WP_011370693.1, ykgO, type B 50S ribosomal protein L36 | -4.53 |
| 7 | WP_029938609.1, ribulose bisphotase carboxylase small subunit | -7.27 | WP_127119115.1, assimilatory sulfite reductase (NADPH) flavoprotein subunit | -5.67 | WP_029939613.1, co.chaperone GroES | -4.49 |
| 8 | WP_127120281.1, cytochrome b | -7.06 | WP_029939088.1, cybH, Ni/Fe-hydrogenase, b-type cytochrome subunit | -5.65 | WP_127119923.1, grpE, nucleotide exchange factor GrpE | -4.40 |
| 9 | WP_029938597.1, BMC domain-containing protein | -6.99 | WP_029939062.1, phosphoadenylyl-sulfate reductase | -5.64 | WP_029939121.1, flagellar protein FliS | -4.38 |
| 10 | WP_127119402.1, ferritin-like domain-containing protein | -6.75 | WP_237260355.1, SLC13 family permease | -5.01 | WP_081836833.1, rpmB, 50S ribosoml protein L28 | -4.29 |
| 11 | WP_127119405.1, rdgB, RdgB/HAM family non-canonical purine NTP pyrophosphatase | -6.62 | WP_029939085.1, HyaD/HydB family dehydrogenase maturation endopeptidase | -4.49 | WP_127119399.1, TetR/AcrR family transcriptional factor | -4.25 |
| 12 | WP_029938870.1, rpmG, 50S ribosomal protein L33 | -5.95 | WP_029939405.1, glutathione peroxidase | -4.23 | WP_225972281.1, hypothetical protein | -4.10 |
| 13 | WP_029938604.1, BMC domain-containing protein | -5.95 | WP_029939084.1, HypC/HybG/HupF family hydrogenase formation chaperone | -4.18 | WP_127119275.1, permease | -4.07 |
| 14 | WP_029938605.1, carboxysome peptide B | -5.80 | WP_029939090.1, YeeE/YedE thiosulfate transporter family protein | -3.89 | WP_127119276.1, MTH895/ArsE family thioredoxin-like protein | -4.03 |
| 15 | WP_127119767.1, MliC family protein | -5.78 | WP_127119032.1, TolC family protein | -3.74 | WP_068650448.1, peptidylprolyl isomerase | -4.00 |
| 16 | WP_127119404.1, hypothetical protein | -5.75 | WP_127119075.1, zigA, zinc metallochaperone GTPase ZigA | -3.70 | WP_029938503.1, exbB, TonB-system energzier ExbB | -3.98 |
| 17 | WP_029939715.1, form I ribulose bisphosbate carboxylase large subunit | -5.67 | WP_051673416.1, PotD/PotF family extracellular solute-binding protein | -3.55 | WP_029938500.1, RDD family protein | -3.95 |
| 18 | WP_127120183.1, speD, adenosylmethionine decarboxylase | -5.57 | WP_127119053.1, metE, 5-methyltetrahydropteroyltriglutamate-homocysteine S-methyltransferase | -3.53 | WP_127119347.1, twin-arginine translocation signal domain-containing protein | -3.94 |
| 19 | WP_029939716.1, ribulose bisphosphate carboxylase small subunit | -5.50 | WP_029939378.1, YifB family protein | -3.52 | WP_127120183.1, speD, adenosylmethionine decarboxylase | -3.91 |
| 20 | WP_029938601.1, 4a-hydroxytetrahydrobiopterin dehydratase | -5.24 | WP_127119285.1, protein glxC | -3.50 | WP_127119334.1, sugar phosphate isomerase/epimerase | -3.91 |
| 21 | WP_029939717.1, CbbQ/NirQ/NorQ/GpvN family protein | -5.13 | WP_029939070.1, TonB-dependent receptor | -3.50 | WP_029938628.1, RrF2 family transcriptional regulator | -3.86 |
| 22 | WP_081836833.1, rpmB, 50S ribosomal protein L28 | -5.00 | WP_029939037.1, FMN-dependent NADH-azoreductase | -3.48 | WP_029938870.1, rpmG, 50S ribosomal protein L33 | -3.80 |
| 23 | WP_029938503.1, exbB, TonB-system energizer ExbB | -4.97 | WP_127119276.1, MTH895/ArsE family thioredoxin-like protein | -3.40 | WP_024852172.1, rpmH, 50S ribosomal protein L34 | -3.79 |
| 24 | WP_029938870.1, rpmG, 50S ribosomal protein L33 | -4.88 | WP_127120309.1, gorA, glutathione-disulfide reductase | -3.36 | WP_011370920.1, P-II family nitrogen regulator | -3.79 |
| 25 | WP_127119763.1, TfoX/Sxy family protein | -4.81 | WP_029939071.1, hypothetical protein | -3.28 | WP_051673559.1, ABC trasporter ATP binding protein | -3.78 |

***Table S3:*** *Composition of the media used for cultivation of Hydrogenovibrio under different conditions.*

|  | **Compound** | **MJ [mM]** | **T-ASW [mM]** | **ASW [mM]** |
| --- | --- | --- | --- | --- |
| medium | NaCl (MW: 58.44) | 513.35 | 430.01 | 470.57 |
|  | K_2_HPO_4_ (MW: 174.18) | 0.80 | 3.10 | 0.29 |
|  | CaCl_2_x2H_2_O (MW: 147.01) | 0.95 | 2.00 | 9.52 |
|  | NH_4_Cl (MW: 53.49) | 4.67 | / | 18.70 |
|  | MgSO_4_x7H_2_O (MW: 246.47) | 13.79 | 6.10 | 27,51 |
|  | MgCl_2_x6H_2_O (MW: 203.30) | 20.56 | / | 26.46 |
|  | KCl (MW: 74.55) | 4.43 | / | 9.66 |
|  | Fe(NH_4_)_2_(SO_4_)_2_x6H_2_O (MW: 482.19) | 0.03 | / | / |
|  | NiCl_2_x6H_2_O (MW: 237.69) | 0.0021 | / | / |
|  | Na_2_SeO_3_x5H_2_O (MW: 263.01) | 0.0019 | / | / |
|  | NaHCO_3_ (MW: 84.01) | 11.90 | 2.40 | 10.00 |
|  | Na_2_S_2_O_3_x5H_2_O (MW: 237.69) | / | 40.01 | / |
|  | Phenolred (MW: 354.38) | / | 0.03 | / |
|  | (NH_4_)_2_SO_4_ (MW: 132.14) | / | 7.60 | / |
|  | Tris (MW: 121.14) | / | 20.00 | / |
|  | HEPES (MW: 238.30) | / | 9.9912 | / |
|  | Resazurin (MW: 251.17) | 0.004 |  | / |
| trace element solutions | Nitrilo acetic acid (MW: 191.14) | 0.0785 | / | 0.067 |
|  | MgSO_4_x7H_2_O (MW: 246.47) | 0.1217 | / | / |
|  | MnSO_4_xH_2_O (MW: 169.01) | 0.0296 | / | / |
|  | NaCl (MW: 58.44) | 0.1711 | / | / |
|  | FeSO_4_x7H_2_O (MW: 278.01) | 0.0036 | / | / |
|  | CoSO_4_x7H_2_O (MW: 281.10) | 0.0064 | / | / |
|  | CaCl_2_x2H_2_O (MW: 147.01) | 0.0068 | / | / |
|  | ZnSO_4_x7H_2_O (MW: 287.56) | 0.0063 | / | / |
|  | CuSO_4_x5H_2_O (MW: 249.69) | 0.0004 | / | / |
|  | KAl(SO_4_)_2_x12H_2_O (MW: 474.39) | 0.0004 | / | / |
|  | H_3_BO_3_ (MW: 61.83) | 0.0016 | 0.00099 | 0.000098 |
|  | Na_2_MoO_4_x2H_2_O (MW: 241.95) | 0.0004 | 0.00015 | 0.00015 |
|  | NiCl_2_x6H_2_O (MW: 237.69) | 0.0013 | 0.00010 | 0.0001 |
|  | Na_2_SeO_3_x5H_2_O (MW: 237.69) | 0.00001 | / | 0.000025 |
|  | Na_2_-EDTAx2H_2_O (MW: 372.24) | / | 0.01400 | / |
|  | FeCl_2_x4H_2_O (MW: 198.81) | / | 0.00750 | 0.01 |
|  | ZnCl_2_ (MW: 136.30) | / | 0.00050 | 0.00051 |
|  | MnCl_2_x4H_2_O (MW: 197.91) | / | 0.00050 | 0.00051 |
|  | CoCl_2_x6H_2_O (MW: 237.93) | / | 0.00080 | 0.0008 |
|  | CuCl_2_x2H_2_O (MW: 170.48) | / | 0.00010 | 0.000012 |
|  | Na_2_WO_4_x2H_2_O (MW: 329.85) | / | / | 0.000024 |
| vitamin solution | Biotin (MW: 244.31) | 0.0001 | / | 0.0000082 |
|  | Folic acid (MW: 441.40) | 0.0000 | / | / |
|  | Pyridoxine-HCl (MW: 205.64) | 0.0005 | / | / |
|  | Thiamine-HClx2H2O (MW: 373.30) | 0.0001 | / | 0,000027 |
|  | Riboflavin (MW: 376.36) | 0.0001 | / | / |
|  | Nicotinic acid (MW: 123.11) | 0.0004 | / | 0.00016 |
|  | D-Ca-panthothenate (MW: 238.27) | 0.0002 | / | 0.000021 |
|  | Vitamin B12 (MW: 1355.37) | 0.000001 | / | 0.0000074 |
|  | p-Aminobenzoic acid (MW: 359.37) | 0.0004 | / | 0.000028 |
|  | Lipoic acid (MW: 206.33) | 0.0002 | / | / |
|  | Pyridoxamin-dihydrochlorid (MW: 241.11) | / | / | 0.00021 |

***Table S4:*** *Overview of genes up-and downregulated in comparison amongst the different growth conditions*

|  | S_2_O_3_^2-^ vs. H_2_ | FeCl_2_ vs. H_2_ | FeCl_2_ vs. S_2_O_3_^2-^ |
| --- | --- | --- | --- |
| Genes upregulated | 221 | 433 | 516 |
| Genes downregulated | 282 | 508 | 575 |

**Table S5:** Calculations of environmental rates of C-fixation and H_2_, Fe(II) and S_2_O_3_^2-^ oxidation. Everything marked in yellow are our own measurements. Everything marked in red is taken from literature as stated in the manuscript. All other values are calculated based on our measurements and the values from literature.

**Tabelle S6**: Overview of the samples used for isolation of the three strains. More information on the location of the vent sites, including maps and pictures of the sampling as well as fluid geochemistry can be found in Adam et al.^11^.

| **Strain** | **Sample** | **Vent field**  **(VF)** | **Sampling device** | **In-situ temperature** | **Material sampled** |
| --- | --- | --- | --- | --- | --- |
| 040 | 040 KIPS C/D | VF4, CIR | KIPS | 14°C | Hydrothermal fluid |
| 083 | 083 KIPS A/B | VF1, SEIR | KIPS | 31°C | Hydrothermal fluid |
| 104 | 104 KIPS C/D | VF2, SEIR | KIPS | 11.6°C | Hydrothermal fluid |

**References:**

1. Qin, H., Guan, X., Bandstra, J. Z., Johnson, R. L. & Tratnyek, P. G. Modeling the Kinetics of Hydrogen Formation by Zerovalent Iron: Effects of Sulfidation on Micro- and Nano-Scale Particles. *Environ. Sci. Technol.* **52**, 13887–13896 (2018).

2. Kvalnes-Krick, K. & Jorns, M. S. Interaction of Tetrahydrofolate and Other Folate Derivatives with Bacterial Sarcosine Oxidase. *Biochemistry* **26**, 7391–7395 (1987).

3. Wagner, M. A. & Jorns, M. S. Folate Utilization by Monomeric versus Heterotetrameric Sarcosine Oxidases. *Arch. Biochem. Biophys.* **342**, 176–181 (1997).

4. Lahham, M., Jha, S., Goj, D., Macheroux, P. & Wallner, S. The family of sarcosine oxidases: Same reaction, different products. *Arch. Biochem. Biophys.* **704**, 108868 (2021).

5. Yu, Q. *et al.* The Rhodanese PspE Converts Thiosulfate to Cellular Sulfane Sulfur in Escherichia coli. *Antioxidants* **12**, (2023).

6. Tsai, Y.-C. C., Lapina, M. C., Bhushan, S. & Mueller-Cajar, O. Identification and characterization of multiple rubisco activases in chemoautotrophic bacteria. *Nat. Commun.* **6**, 8883 (2015).

7. Tsai, Y. C. C., Liew, L., Guo, Z., Liu, D. & Mueller-Cajar, O. The CbbQO-type rubisco activases encoded in carboxysome gene clusters can activate carboxysomal form IA rubiscos. *J. Biol. Chem.* **298**, 101476 (2022).

8. Böhnke, S. & Perner, M. Unraveling RubisCO Form I and Form II Regulation in an Uncultured Organism from a Deep-Sea Hydrothermal Vent via Metagenomic and Mutagenesis Studies. *Front. Microbiol.* **8**, 1303 (2017).

9. Solmi, L. *et al.* Polyamine-mediated mechanisms contribute to oxidative stress tolerance in Pseudomonas syringae. *Sci. Rep.* **13**, 1–16 (2023).

10. Garber, A. I. *et al.* FeGenie: A Comprehensive Tool for the Identification of Iron Genes and Iron Gene Neighborhoods in Genome and Metagenome Assemblies. *Frontiers in Microbiology* **11**, 37 (2020).

11. Adam-Beyer, N. *et al.* Microbial ecosystem assessment and hydrogen oxidation potential of newly discovered vent systems from the Central and South-East Indian Ridge. *Front. Microbiol.* **14**, (2023).
